# PACAP38 and PACAP6-38 Exert Cytotoxic Activity Against Human Retinoblastoma Y79 Cells

**DOI:** 10.1007/s12031-014-0248-0

**Published:** 2014-02-11

**Authors:** Jakub Wojcieszak, Jolanta B. Zawilska

**Affiliations:** Department of Pharmacodynamics, Medical University of Lodz, Muszynskiego 1 Street, Lodz, 90-151 Poland

**Keywords:** PACAP, Retinoblastoma, Y79 cells, Cytotoxicity, PAC_1_ receptor

## Abstract

Pituitary adenylate cyclase activating polypeptide (PACAP) is a multifunctional neuropeptide expression of which has been found in various tumors of the brain and peripheral organs. Despite numerous studies, the exact role the peptide plays in the development and progression of tumors is not fully understood. In the present study, we investigated the effect of PACAP on human retinoblastoma Y79 cell viability. We found that both PACAP38 and PACAP6-38, a selective PAC_1_ receptor antagonist, did not affect Y79 cell viability at nanomolar concentrations, but when used at 1–5 μM potently reduced cell survival in a dose-dependent manner. PACAP27 and maxadilan, a high affinity agonist of PAC_1_ receptors, had negligible effects. Two membrane-penetrating analogs of PACAP38 inactive at PAC_1_/VPAC receptors, [Disc^6^]PACAP38 and FITC-Ahx-PACAP11-38, also decreased viability of Y79 cells, albeit with lower potency than PACAP38. The cytotoxic effect of PACAP38 was augmented by p38, MEK1/2, and JNK inhibitors, indicating that high concentrations of the peptide might decrease the activity of these kinases, leading to cell death. It is suggested that the cytotoxic activity of PACAP38 and PACAP6-38 against human retinoblastoma Y79 cell line may result from their interaction with target sites other than PAC_1_ and VPAC receptors, but this is yet unknown.

## Introduction

Pituitary adenylate cyclase activating polypeptide (PACAP) is a multifunctional neuropeptide which belongs to the secretin/glucagon/vasoactive intestinal peptide (VIP)/growth hormone releasing factor peptides superfamily (Vaudry et al. 2009). It was isolated for the first time from an extract of ovine hypothalamus and named after its ability to stimulate adenylate cyclase in cultured rat anterior pituitary cells (Miyata et al. 1989). Endogenously, PACAP exists in two forms, the dominating PACAP38 and *C*-truncated PACAP27 which shares 68 % of structural similarity with VIP (Vaudry et al. 2009). Consistent with its widespread distribution in the central nervous system and peripheral tissues, PACAP has been found to exert pleiotropic physiological functions acting as a hormone, a neurohormone, a neurotransmitter, and a trophic factor. The peptide has been shown to be involved in modulation of neurotransmitter release, neuroprotection, vasodilation, bronchodilation, activation of intestinal motility, increase of insulin and histamine secretion, as well as stimulation of cell multiplication and/or differentiation (Vaudry et al. 2009).

PACAP38 and PACAP27 exert their biological activity by stimulating specific membrane bound G protein-coupled receptors, namely PAC_1_, VPAC_1_ and VPAC_2_. PAC_1_ receptor exhibits much higher affinity to both forms of PACAP than to VIP, whereas VPAC_1_ and VPAC_2_ receptors have similar affinity for VIP and PACAPs. Several splice variants of PAC_1_ receptor have been identified and characterized. They express marked differences not only in terms of tissue localization but also in second messengers coupling (Vaudry et al. 2009). PACAP can also bind to other target sites, such as CART (Lin et al. 2011) and secretin receptors (Felley et al. 1992). Recently, it has been demonstrated that PACAP and its synthetic derivatives can cross plasma membrane via a receptor-independent manner: by a direct translocation, endocytosis through clathrin-coated pits and macropinocytosis or by a clathrin-independent caveolar endocytosis (Doan et al. 2012a, 2012b). Furthermore, PACAP has been found to evoke re-dimerization of PAC_1_ receptor on the nucleus and, when used at high concentrations, to cause translocation of PAC_1_ dimers into nucleus accompanied with increased cAMP levels in the nuclear fraction (Yu et al. 2013).

Expression of PACAP has been found in various tumors of the brain (gliomas, neuroblastomas) and peripheral organs, such as pheochromocytomas, pituitary, pancreas, and ovarian carcinomas (Vaudry et al. 2009). Despite numerous studies, the exact role the peptide plays in the development and progression of tumors is not fully understood. It has been demonstrated that depending on the cell line, PACAP can increase or decrease cancer cell viability. Thus, PACAP38 stimulated proliferation of rat C6 glioma cells (Sokołowska and Nowak 2008) and prevented apoptosis of androgen-independent prostate cancer PC-3 cells (Gutiérrez-Cañas et al. 2003). On the other hand, PACAP27 reduced proliferation of two human colonic tumor cell lines: DLD-1 and Caco-2 (Lelièvre et al. 1998), and PACAP38 inhibited proliferation of primary medulloblastoma-derived tumor spheres (Cohen et al. 2010). Furthermore, an increased colorectal tumor incidence was observed in PACAP knockout mice (Nemetz et al. 2008). Although the presence of specific functional PAC_1_ receptors has been demonstrated in human retinoblastoma Y79 cell line (Olianas et al. 1996; Dautzenberg et al. 1999), a potential role PACAP might play in this type of tumor remains to be elucidated. Therefore, the aim of the current work was to investigate whether PACAP affected viability of Y79 cells and to shed some light on mechanism(s) that may be involved in this action.

## Materials and Methods

### Reagents

PACAP27, PACAP38, and PACAP6-38 were purchased from PolyPeptide Laboratories (Strasbourg, France). Maxadilan was purchased from Bachem AG (Bubendorf, Switzerland). [Disc^6^]PACAP38, FITC-Ahx-PACAP11-38, FITC-Ahx-PACAP28-38, and FITC-Ahx-TAT(48-60) were generous gifts from Dr. Myriam Letourneau and Dr. Alain Fournier from Laboratoire d’Études Moléculaires et Pharmacologiques des Peptides, INRS–Institut Armand-Frappier, Université du Québec, Canada. MTT (3-(4,5-dimethyl-2-thiazolyl)-2,5-diphenyl-2*H*-tetrazolium bromide), SP600125 (1,9-pyrazoloanthrone), U0126 (1,4-diamino-2,3-dicyano-1,4-bis(o-aminophenylmercapto)butadiene), SB203580 (4-(4-fluorophenyl)-2-(4-methylsulfinylphenyl)-5-(4-pyridyl)1*H*-imidazole), GF 109203X (3-(*N*-[dimethylamino]propyl-3-indolyl)-4-(3-indolyl)maleimide), and H89 (*N*-[2-(p-bromocinnamylamino)ethyl]-5-isoquinoline sulfonamide) were purchased from Sigma-Aldrich (Poznań, Poland). RPMI 1640 medium, heat inactivated fetal bovine serum, penicillin, and streptomycin were purchased from Life Technologies (Warsaw, Poland).

### Cell Culture

Y79 cells, purchased from the European Collection of Cell Cultures (ECACC, Porton Down, UK), were cultured in RPMI 1640 medium supplemented with 10 % fetal bovine serum and penicillin-streptomycin (10,000 U/ml) at 37 °C in a humidified atmosphere with 5 % CO_2_. Upon reaching the density of approx. 900,000 cells/ml, cells were passaged into a new flask or resuspended in a fresh, serum-free medium and seeded into 96-well plates at a density of 20,000 cells/well in 80 μl of medium. Following overnight incubation, solutions of PACAP27, PACAP38, PACAP6-38, maxadilan, [Disc^6^]PACAP38, FITC-Ahx-PACAP11-38, FITC-Ahx-PACAP28-38 or FITC-Ahx-TAT(48-60) were added into the wells. After incubation for 24 h, measurements of cell viability were conducted using MTT and LDHe assays.

### MTT Assay

Cell viability and mitochondrial function were measured by assessment of 3-(4,5-dimethyl-2-thiazolyl)-2,5-diphenyl-2*H*-tetrazolium bromide (MTT) reduction to formazan by mitochondrial dehydrogenases. Following exposure to the tested compounds, solution of MTT (0.5 mg/ml) was added and cells were incubated for 3 h at 37 °C. Formazan crystals were solubilized in isopropanol acidified with 0.04 N HCl and absorbance, proportional to the number of viable cells, was measured at 570 nm using Bio-Rad microplate reader model 680.

### LDHe Assay

Cellular membrane integrity was assessed by extracellular lactate dehydrogenase activity measurement using LDH Cytotoxicity Assay (ScienCell Research Laboratories, Carlsbad, CA, USA), following manufacturer’s instructions.

### Data Analysis

Data were expressed as the mean ± standard error of the mean (SEM) values and were analyzed for statistical significance using one-way ANOVA, followed by post hoc Student-Newman-Keul’s test using InStat version 3.06 for Windows (GraphPad, San Diego, CA, USA).

## Results

### PACAP38 and PACAP6-38 are Cytotoxic to Y79 Cells

**Fig. 1:**
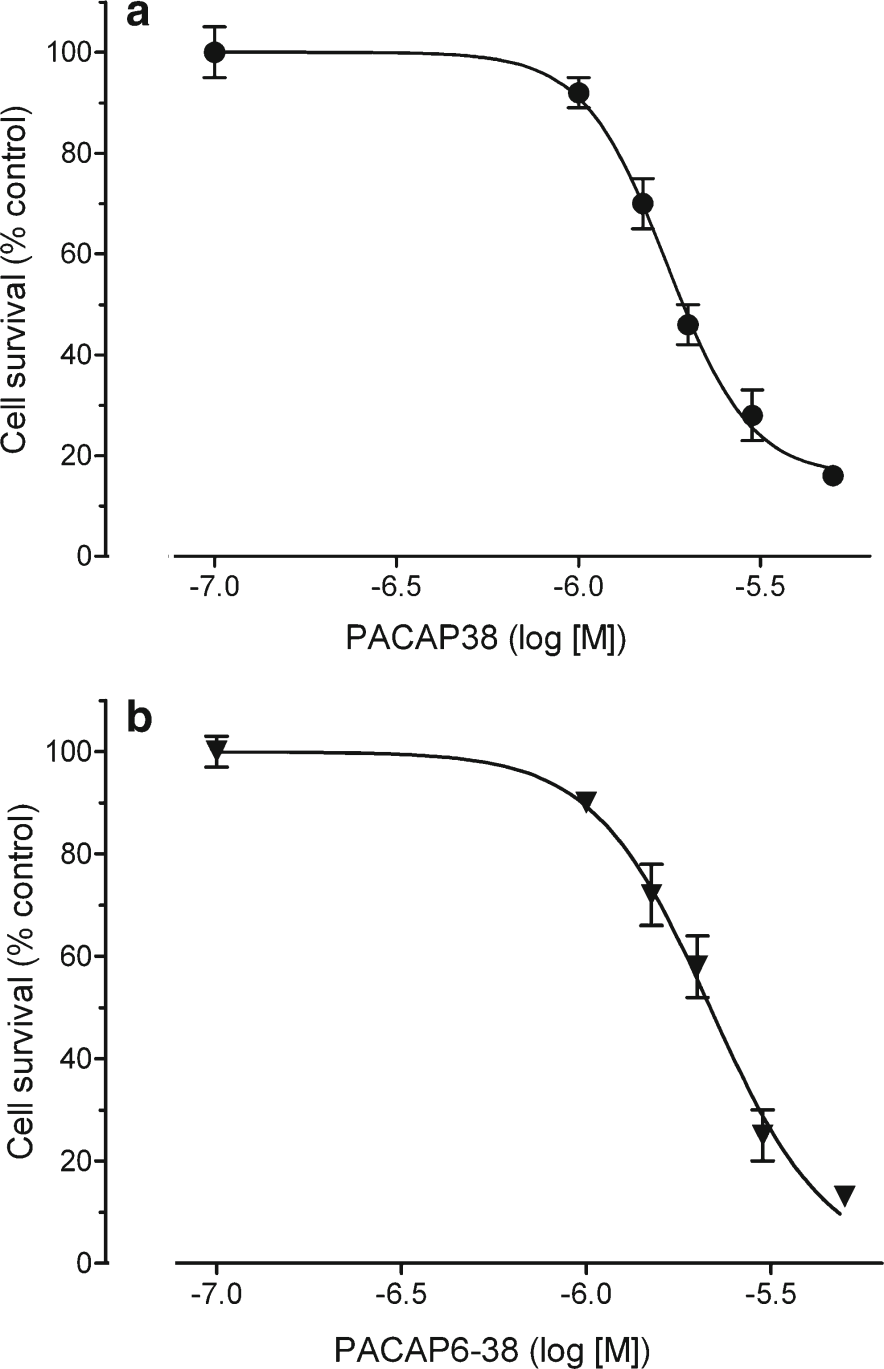
Concentration-dependent effect of PACAP38 (panel **a**) and PACAP6-38 (panel **b**) on viability of Y79 cells. Cells were incubated with the peptides (0.1–5 μM) for 24 h and cell viability was analyzed by MTT test. Data are presented as mean ± SEM of 6–12 values per group and expressed as a percentage of the respective control

**Fig. 2:**
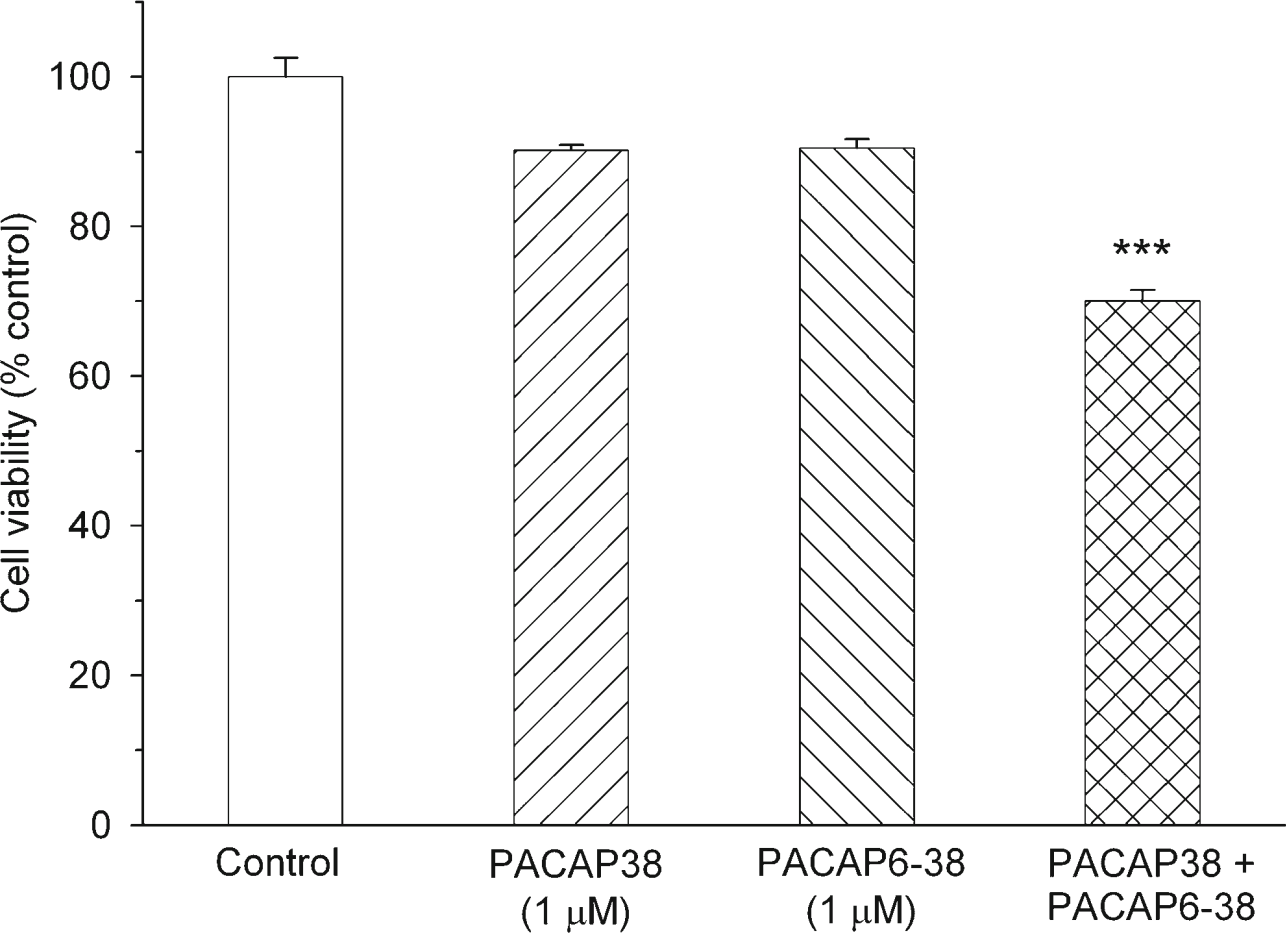
The additive effect of PACAP38 and PACAP6-38 on viability of Y79 cells. Cells were incubated with 1 μM of the peptides for 24 h and cell viability was analyzed by MTT test. Data are presented as mean ± SEM of 6–12 values per group and expressed as a percentage of the respective control. ***p < 0.001 vs. control

**Fig. 3:**
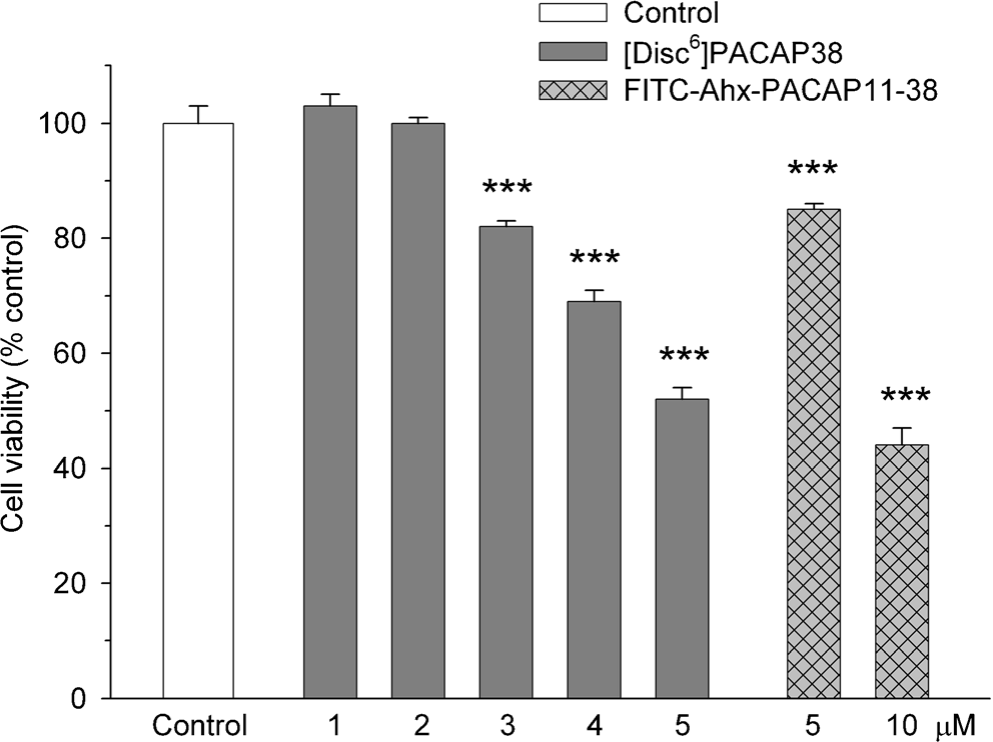
Effect of [Disc^6^]PACAP38 and FITC-Ahx-PACAP11-38 on viability of Y79 cells. Cells were incubated with the peptides for 24 h and cell viability was analyzed by MTT test. Data are presented as mean ± SEM of 6–12 values per group and expressed as a percentage of the respective control.***p < 0.001 vs. control

Y79 cells were challenged with various peptides and cell viability, and mitochondrial function was measured by MTT test. Twenty-four-hour incubation of the cells with nanomolar (0.1–100 nM) concentrations of PACAP38 had no effect on their viability (data not shown). When the peptide was used in higher concentrations (1–5 μM) it produced a dose-dependent decrease in cell viability, with a calculated IC_50_ = 1.7 μM (Fig. 1a). The cytotoxic effect of PACAP38 was not abolished by a PAC_1_ receptor antagonist, PACAP6-38. In fact, PACAP6-38 (1–5 μM) also produced a concentration-dependent reduction of Y79 cell viability with a similar potency and IC_50_ = 2.1 μM (Fig. 1b). The suppressive effects of both peptides on Y79 cell viability were additive (Fig. 2). On the contrary, incubation of Y79 cells with PACAP27 (0.1–5 μM) and maxadilan (1 and 2 μM), a high affinity selective agonist of PAC_1_ receptors, did not significantly affect their viability (data not shown). Two membrane-penetrating analogs of PACAP38 inactive at PAC_1_/VPAC receptors, [Disc^6^]PACAP38 and FITC-Ahx-PACAP11-38, decreased viability of Y79 cells, albeit with lower potency than PACAP38 (Fig. 3). Another cell penetrating peptide, FITC-Ahx-TAT48-60, which has no structure similarity to PACAP, and membrane non-penetrating *C*-terminal fragment of PACAP, FITC-Ahx-PACAP28-38, used at a 10 μM concentration were inactive (data not shown).

**Fig. 4:**
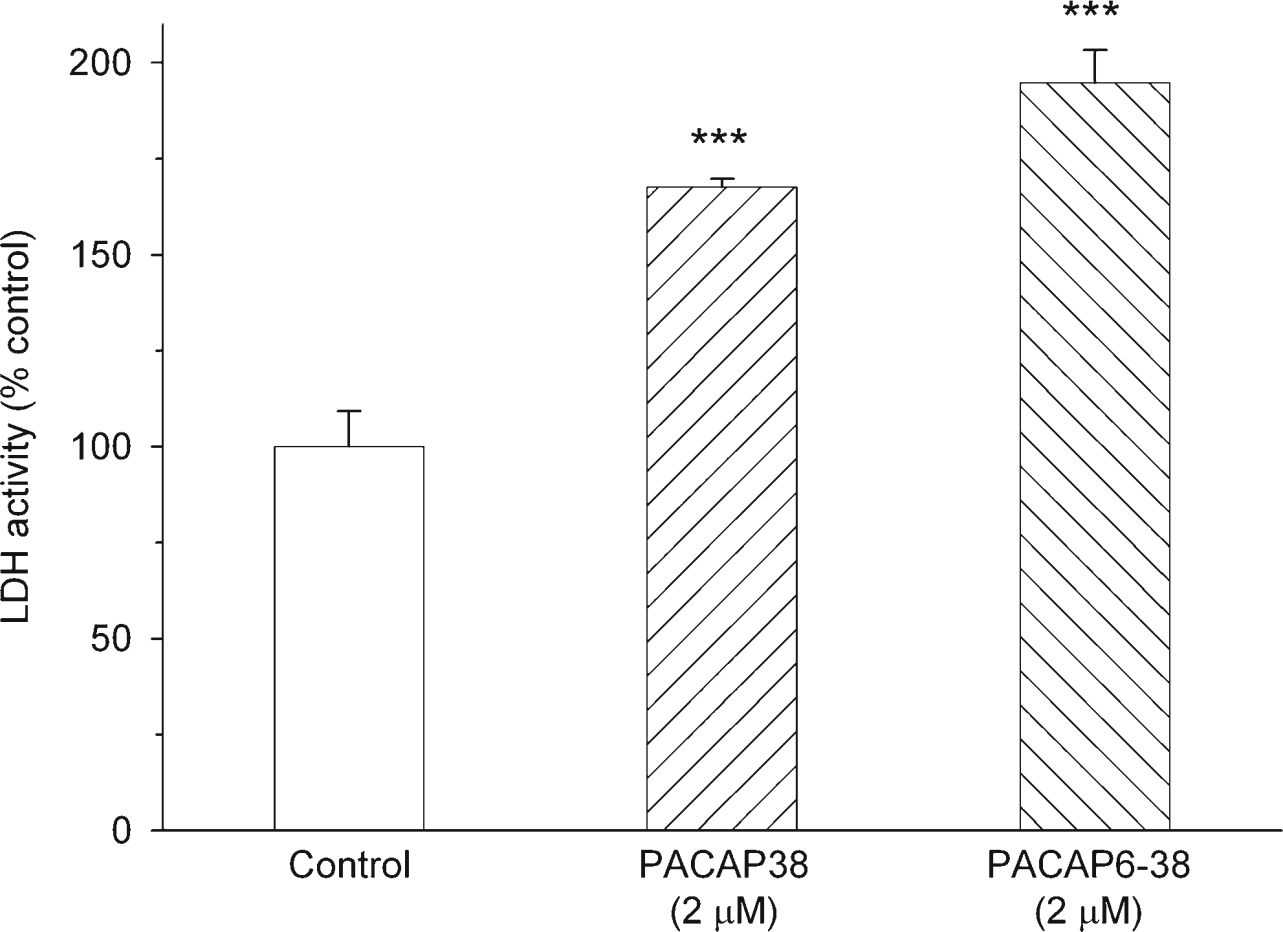
Effect of PACAP38 and PACAP6-38 on viability of Y79 cells. Cells were incubated with 2 μM of the peptides for 24 h and cell viability was analyzed by assessment of cell membrane integrity using LDHe test. Data are presented as mean ± SEM of 6–12 values per group and expressed as a percentage of the respective control. ***p < 0.001 vs. control

In another set of experiments, we examined effects of PACAP38 and PACAP6-38 on Y79 cell viability by measuring extracellular activity of lactate dehydrogenase (LDH). LDH is a cytosolic enzyme present in most eukaryotic cells, which is released into the culture medium upon cell death due to the damage of plasma membrane. Incubation of Y79 cells with PACAP38 and PACAP6-38, both at 2 μM, for 24 h resulted in a significant increase of extracellular LDH activity by 68 and 95 %, respectively (Fig. 4).

### Effects of Signal Transduction Inhibitors on PACAP38-Induced Decrease in Y79 Cell Viability

**Fig. 5:**
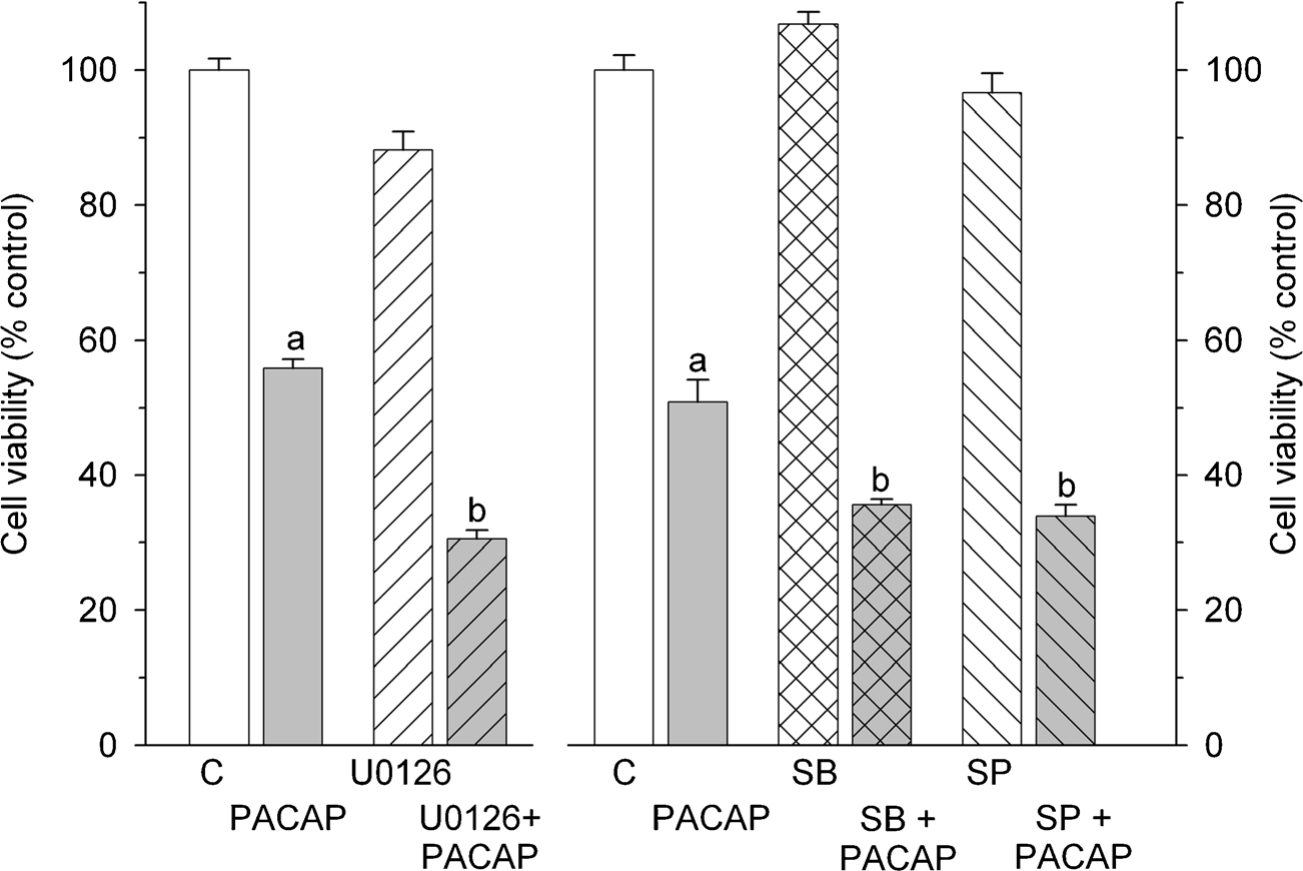
Effect of signal transduction inhibitors on the PACAP38-induced decrease in viability of Y79 cells. Following 1 h pretreatment with U0126 (MEK1/2 inhibitor; 10 μM); SB203580 (SB, p38 inhibitor; 10 μM); SP600125 (SP, JNK inhibitor; 10 μM) cells were incubated for 24 h with PACAP38 (2 μM). Cell viability was analyzed by MTT test. Data are presented as mean ± SEM of 6–12 values per group and expressed as a percentage of the respective control (C). *a* indicates *p* < 0.001 vs. control, *b* indicates *p* < 0.001 vs. PACAP38

In order to shed some light on biochemical mechanism(s) underlying the cytotoxic effect of PACAP38 on Y79 cells, cells were preincubated for 1 h with the following signal transduction inhibitors: U0126 (MEK1/2 inhibitor; 10 μM), SB203580 (p38 inhibitor; 10 μM), SP600125 (JNK inhibitor; 10 μM), GF 109203X (PKC inhibitor; 2.5 μM), and H89 (PKA inhibitor; 5 μM). U0126, SB203580, and SP600125 significantly facilitated the PACAP38-induced decrease in Y79 cell survival (Fig. 5), whereas the other tested inhibitors were inactive (data not shown).

## Discussion

Using different experimental approaches (RT-PCR analysis, radioligand binding of ^125^I-PACAP27, and measurement of adenylyl cyclase activity), the presence of functional PAC_1_ receptors has been demonstrated in Y79 human retinoblastoma cells (Olianas et al. 1996; Dautzenberget al. 1999). Despite these findings, a potential role PACAP might play in this type of tumor is at present unknown. In the current study, we analyzed effects of PACAP38 on viability of Y79 human retinoblastoma cell line. Using two complementary methods, namely, MTT test and extracellular LDH assay, we found that the peptide strongly, albeit with a low potency, reduced survival of Y79 cells. Surprisingly, PACAP6-38 not only did not block the effect of PACAP38, but produced reduction of Y79 cell viability with a similar potency to PACAP38, while PACAP27 and maxadilan, a selective high affinity peptidergic agonist of PAC_1_ receptors, had neglible activity. The cytotoxic effect of PACAP38 and PACAP6-38 was characteristic to Y79 cells as they were inactive at two other studied cell types, i.e., neuroblastoma SH-SY5Y and rat cortical astrocytes (Wojcieszak J, unpublished data).

It should be noted that although PACAP6-38 acts as the antagonist of PAC_1_ and VPAC_2_ receptors (Vaudry et al. 2009), there are also reports demonstrating that in certain models this peptide can behave similarly to PACAP38. Thus, in isolated rat tracheae, both peptides inhibited the release of substance P, calcitonin gene-related peptide, and somatostatin evoked by both chemical excitation and electrical field stimulation of capsaicin-sensitive afferents (Reglodi et al. 2008). In human cytotrophoblast cells, PACAP38 and PACAP6-38 stimulated ERK1/2 and JNK phosphorylation, while they both inhibited p38 MAPK phosphorylation (Reglodi et al. 2008). In another study, both PACAP38 and PACAP6-38 caused enhancement of phagocytosis in mouse macrophages with a similar potency, while PACAP6-27 produced less pronounced increase, and the effect PACAP27 was even weaker (Ichinose et al. 1995). In chicken limb bud-derived chondrogenic cells, PACAP38 and PACAP6-38 stimulated cell proliferation and enhanced expression of PAC_1_ receptor, Sox9 protein, and calcineurin (Juhasz et al. 2013).

In our studies, PACAP38 and PACAP6-38 were effective when used at high, micromolar concentrations. Similar data have been recently reported by other authors. Baun et al. (2012) have shown degranulation of rat peritoneal mast cells after incubation with micromolar concentrations of PACAP38. By analogy to our results, PACAP38 and PACAP6-38 produced similar effects, whereas PACAP27 triggered markedly weaker response and maxadilan was inactive (Baun et al. 2012). Furthermore, PACAP38 applied at micromolar concentration increased expression of proinflammatory cytokines, IL-1β and TNF-α, in grass carp head kidney and head kidney leucocytes (Wang et al. 2013).

Several lines of evidence suggest that the cytotoxic effect of PACAP on human retinoblastoma Y79 cells is independent of PAC_1_ and VPAC receptors and might be related to the peptide sequence. Firstly, the calculated IC_50_ values for PACAP38 and PACAP6-38 were around 2 μM, while binding affinity of PACAP38 to PAC_1_/VPAC receptors is within nanomolar range: *K*_*d*_ ≈ 0.5 nM at the full length PAC_1_ receptor, *K*_*d*_ ≈ 1.0 nM at VPAC_1_ and VPAC_2_ receptors, and the affinity of PACAP6-38 for PAC_1_ is approximately 10-fold lower than that of PACAP38 (Bourgault et al. 2009; Vaudry et al. 2009). Secondly, maxadilan, the potent and selective PAC_1_ receptor agonist, and PACAP27, the *C*-truncated form of PACAP38, were inactive. Thirdly, [Disc^6^]PACAP38 and FITC-Ahx-PACAP11-38, the membrane permeable synthetic analogs of PACAP38, produced similar, but less pronounced reduction in human retinoblastoma Y79 cell viability. FITC-Ahx-PACAP11-38 is an inactive analog of PACAP38 obtained by the removal of 10 *N*-terminal amino acids. In the original peptide, these amino acids form a Asx-turn-like motif and N-capping secondary structure responsible for the receptor recognition, selectivity, and activation (Bourgault et al. 2009; Doan et al. 2011, 2012b). [Disc^6^]PACAP38 is another receptor-inactive analog of PACAP38. It is produced by the substitution of Phe^6^ residue essential for the biological activity of PACAP with a conformationally constrained 1,3-dihydro-2*H*-isoindole carboxylic acid (Bourgault et al. 2009). Both analogs conserve helical properties of PACAP38 that underlie the membrane-penetrating activity of the peptide (Doan et al. 2011, 2012b). The fact that PACAP38 and its fragments evoke similar effects in Y79 cells in relatively high concentrations might suggest that their common, shorter metabolite, arising in low quantity, may be responsible for the cytotoxicity, or that the peptide fragment responsible for cytotoxicity is rapidly metabolized. PACAP38 can be metabolized in vivo and in vitro by dipeptidyl peptidase IV (DPP IV), intracellular enzymes and human plasma enzymes, giving rise to shorter fragments, such as PACAP3-38, PACAP5-38, PACAP22-38, PACAP1-21, PACAP1-20, PACAP1-19, PACAP1-37, PACAP1-34, PACAP1-30, and PACAP1-29 (Bourgault et al. 2008; Doan et al. 2012a; Zhu et al. 2003). Metabolites that may be produced from PACAP38 by Y79 cells are at present unknown.

The cytotoxic activity of PACAP38 against human retinoblastoma Y79 cell line might result from its interaction with target sites other than PAC_1_ and VPAC receptors, but this is yet unknown. PACAP38 has been found to have higher ability to cross cell membrane than PACAP27 in three cell lines, CHO-K1, HEK293, and HeLa, with a significant increase of intracellular fraction at micromolar concentration. It has been suggested that the efficacy of peptides to penetrate into the cell may depend on the cell membrane composition or the cell size (Doan et al. 2012a). There is evidence that PACAP can bind to receptors localized inside the cell, i.e., on the nucleus (Doan et al. 2012a; Yu et al. 2013). The fact that high concentrations of both PACAP38 and PACAP6-38 exert similar cytotoxic effects in Y79 cells could be explained by their limited Y79 cell membrane permeability, whereas PACAP27, due to its lower permeability, may not reach sufficient concentration inside the cells. On the other hand, FITC-Ahx-PACAP28-38, inactive in our studies, contains a highly basic *C*-terminal fragment of PACAP38 and does not enter the intracellular compartment, a property that is likely due to the disordered secondary structure of this segment (Doan et al. 2012a).

Neither of signal transduction inhibitors used abolished the effect of PACAP38, an observation indicating that the peptide-induced cell death does not involve activation of PKA, PKC, MEK1/2, p38, and JNK kinases. On the other hand, MEK1/2, p38, and JNK inhibitors augmented PACAP38 cytotoxicity, which may suggest that high concentrations of the peptide inhibit those kinases, leading to the cell death.

In summary, we found that PACAP38 and PACAP6-38 as well as two PACAP38 analogs, [Disc^6^]PACAP38 and FITC-Ahx-PACAP11-38, inactive at PAC_1_/VPAC receptors, produced marked decrease in Y79 cells viability at micromolar concentrations. It is suggested that the cytotoxic activities of the peptides is independent from activation of PAC_1_/VPAC receptors. Further studies are needed in order to define the exact molecular mechanism(s) of PACAP38-induced cytotoxicity in Y79 human retinoblastoma cells.

## Abbreviations

JNK: c-Jun *N*-terminal kinases
LDH: Lactate dehydrogenase
MAPK: Mitogen-activated protein kinase
PKA: Protein kinase A
PKC: Protein kinase C
PACAP: Pituitary adenylate cyclase activating polypeptide
PAC_1_: Selective receptor for PACAP
VIP: Vasoactive intestinal peptide
VPAC_1_: Type 1 receptor for PACAP and VIP
VPAC_2_: Type 2 receptor for PACAP and VIP
